# Halofuginone Attenuates Osteoarthritis by Rescuing Bone Remodeling in Subchondral Bone Through Oral Gavage

**DOI:** 10.3389/fphar.2018.00269

**Published:** 2018-03-27

**Authors:** Wenbo Mu, Boyong Xu, Hairong Ma, Jiao Li, Baochao Ji, Zhendong Zhang, Abdusami Amat, Li Cao

**Affiliations:** ^1^Orthopaedics, First Affiliated Hospital of Xinjiang Medical University, Ürümqi, China; ^2^State Key Laboratory of Pathogenesis, Prevention and Treatment of High Incidence Diseases in Central Asian Xinjiang Key Laboratory of Echinococcosis, Clinical Medical Research Institute, First Affiliated Hospital of Xinjiang Medical University, Ürümqi, China

**Keywords:** osteoarthritis, articular cartilage, subchondral bone, TGF-β1, halofuginone

## Abstract

Osteoarthritis (OA) is a common debilitating joint disorder worldwide without effective medical therapy. Articular cartilage and subchondral bone act in concert as a functional unit with the onset of OA. Halofuginone is an analog of the alkaloid febrifugine extracted from the plant *Dichroa febrifuga*, which has been demonstrated to exert inhibition of SMAD 2/3 phosphorylation downstream of the TGF-β signaling pathway and osteoclastogenesis. To investigate whether halofuginone (HF) alleviates OA after administration by oral gavage, 3-month-old male mice were allocated to the Sham group, vehicle-treated anterior cruciate ligament transection (ACLT) group, and HF-treated ACLT group. The immunostaining analysis indicated that HF reduced the number of matrix metalloproteinase 13 (MMP-13) and collagen X (Col X) positive cells in the articular cartilage. Moreover, HF lowered histologic OA score and prevented articular cartilage degeneration. The micro-computed tomography (μCT) scan showed that HF maintained the subchondral bone microarchitecture, demonstrated by the restoration of bone volume fraction (BV/TV), subchondral bone plate thickness (SBP.Th.), and trabecular pattern factor (Tb.Pf) to a level comparable to that of the Sham group. Immunostaining for CD31 and μCT based angiography showed that the number and volume of vessels in subchondral bone was restored by HF. HF administered by oral gavage recoupled bone remodeling and inhibited aberrant angiogenesis in the subchondral bone, further slowed the progression of OA. Therefore, HF administered by oral gavage could be a potential therapy for OA.

## Introduction

Osteoarthritis (OA) is the most common degenerative condition of the weight-bearing joints, characterized by pain and loss of function (McAlindon et al., 2014). Date from the Third National Health and Nutrition Examination Survey in USA showed that the prevalence of knee joint OA was 12.1% (Pereira et al., 2011). In Asia, knee joint OA affects an estimated 5.7 and 4.4% of men and 10.3 and 19.2% of women in China and Korea respectively (Tang et al., 2015; Lee and Kim, 2017). It's been estimated that the cost of OA treatment is up to 25–50% of a country's GDP (Puig-Junoy and Ruiz Zamora, 2015). The annual average direct cost of OA management varies from $1,442 to $21,335 per person (Xie et al., 2016). Currently, effective disease-modifying therapy for OA is still lacking, leaving pain management and joint replacement as the last option for end-stage OA (Bijlsma et al., 2011; Carr et al., 2012). The pathogenesis of OA is not well understood (van den Berg, 2011), and an improved understanding is greatly needed to develop preventative and effective therapeutic interventions for early-stage OA.

Although maintenance of the articular cartilage is the primary concern of OA treatment, this disease affects the entire joint (Loeser et al., 2012; Findlay and Kuliwaba, 2016). The characteristic pathological changes in OA include articular cartilage degeneration, subchondral bone sclerosis, inflammation, and osteophyte formation (Goldring and Goldring, 2016; Robinson et al., 2016). The articular cartilage and subchondral bone act as a functional unit that transfers the load during weight-bearing and joint motion (Madry et al., 2010; Lories and Luyten, 2011). The subchondral bone, which plays a pivotal role in the initiation and progression of OA, exhibits changes prior to those seen in the articular cartilage (Goldring and Goldring, 2007; Loeser et al., 2012). The subchondral bone is located immediately beneath the CC and can adapt its structural and functional property during modeling and remodeling in response to mechanical stress (Burr and Gallant, 2012).

The temporally and spatially regulated osteoclast and osteoblast activity guarantee the integrity of the subchondral bone (Cao, 2011). Osteoclasts remove bone and, thereby, form the bone marrow microenvironment, which is followed by targeted osteogenesis and angiogenesis for subsequent osteoblast bone formation (Tang et al., 2009). Alterations in the underlying subchondral bone that eventually affect the overlying articular cartilage occur under certain conditions such as ligament injury, overweight individuals, and age-related weakening of muscle strength (Burr and Radin, 2003).

Transforming growth factor β1 (TGF-β1) is a polypeptide member of the TGF superfamily of cytokines and (de Caestecker, 2004) high levels have detrimental effects on adult joints. Itayem et al. (1999) reported that injecting TGF-β1 into the knee joints of adult rats resulted in the onset of OA. Maeda et al. (2011) found that elevated TGF-β1 levels were harmful to the tendon. Therefore, maintaining physiological levels of TGF-β1 in the subchondral bone is crucial. TGF-β1 has been noted to be an important coupling factor of bone resorption and formation and is activated unduly by elevated osteoclast bone resorption when mechanical loading is altered. High levels of active TGF-β1 in subchondral bone uncouples bone remodeling, which further adversely affects the overlying articular cartilage (Zhen et al., 2013). Besides, elevated TGF-β1 activity has been shown to promote angiogenesis and abnormal angiogenesis in subchondral bone is a known pathological feature of OA (Arnoldi et al., 1972; Cunha and Pietras, 2011). Therefore, targeting excessively elevated TGF-β1 signaling in the subchondral bone attenuates the progression of OA (Xie et al., 2016).

Halofuginone (HF) is an analog of the alkaloid febrifugine, which was originally isolated from the plant *Dichroa febrifuga* (Pines and Nagler, 1998) and it is used in commercial poultry production worldwide (Pinion et al., 1995). Increasing attention has been focused on this small molecule because of its beneficial biological activity. HF has been shown effective in treating fibrotic disease, e.g., chronic graft-vs.-host disease and AIDS-related Kaposi sarcoma in human clinical trials (Pines et al., 2003; Koon et al., 2011). It is reported to play an important role in inhibiting fibrosis and the transition of fibroblasts to myofibroblasts by inhibiting SMAD 2/3 phosphorylation downstream of the TGF-β signaling pathway (Pines, 2008, 2014). Besides, HF has been shown to exhibit antiangiogenesis effect from *in vivo* and *in vitro* study through inhibiting sequential events involved in the angiogenic cascade, such as MMP-2 expression, basement membrane invasion, capillary tube formation and deposition of sub-endothelial ECM (Abramovitch et al., 1999; Elkin et al., 2000; Spector et al., 2010). Moreover, HF has also been demonstrated to inhibit TH17 cell differentiation by activating the Amino Acid Starvation Response (Sundrud et al., 2009). The intraperitoneal administration of HF has been shown to attenuate OA progression in one instance by targeting elevated subchondral bone TGF-β1 activity in a rodent OA model (Cui et al., 2016). The oral route of administration is a common, convenient, and noninvasive treatment procedure used in scientific experimentation, although it is not always as efficient as other more invasive ways. The aim of the present study is to investigate the potential attenuation of OA progression by HF following administration by oral gavage in a rodent anterior cruciate ligament transection (ACLT) model, which may provide evidence to support the oral route as an alternative route of HF administration for potential clinical application.

## Materials and methods

### Mice

Three-month-old male C57BL/6 mice were purchased from Vital River and maintained in an animal room on a 12-h light/dark cycle with a temperature and humidity of 25 ± 2°C and 55%, respectively. The mice were provided with food and water *ad libitum* and subsequently divided into the Sham group, vehicle-, and HF-treated OA groups (*n* = 6–8 per group). Before the surgery to establish the OA model, the mice were anesthetized using a combination of ketamine and xylazine (80/10 mg/kg) administered intraperitoneally. Following a parapatellar incision, the anterior cruciate ligament (ACL) of the right knee was transected using a pair of microscissors to establish the OA model. Then, the joint capsule and skin were sutured separately. For the Sham group, after anesthesia, a sham operation was performed on each mouse, which is, making the parapatellar incision in the right knee joint to expose the ACL, followed by the joint capsule and skin sutured separately without transecting the ACL. After the surgery, the mice were exposed to heat and were monitored until they recovered from the anesthesia. The mice were checked daily postoperatively to monitor their general health, pain, discomfort, and development of infections. To select the HF dose, a single-dose acute toxicity test was performed in C57BL/6 male mice of 3-month-old and the median lethal dose (LD_50_) was calculated by the method of Bliss (Pinion et al., 1995). HF hydrobromide (17395-31-2, Watson & Noke) was administered at single doses of 1.068, 1.424, 1.898, 2.531, 3.375, 4.5, and 6 mg/kg body weight by oral gavage, followed by a 14-day observation (*n* = 13/group). After the dose range selection, HF hydrobromide at three different doses or an equivalent volume of distilled water was administered by oral gavage every other day for 30 days after postoperative day 2. The mice were euthanized 30 and 60 days postoperatively. All the animal experiment protocols were reviewed and approved by the Institutional Animal Care and Use Committee of First Affiliated Hospital of Xinjiang Medical University.

### Histochemistry, immunohistochemistry, and histomorphometry

Following euthanasia, the right knee joints of the mice were harvested, fixed in 10% buffered formalin for 24 h, and then the specimens were decalcified in 10% ethylene diamine tetraacetic acid (EDTA, pH 7.3) for 21 days, followed by embedment in paraffin (Leica), sagittally. Then, 4-μm-thick serial sections of the medial compartment of the right knee joint were processed for Safranin O—Fast Green, and hematoxylin and eosin (H&E) staining. The tidemark was defined as the boundary between the non-calcified and calcified cartilage (CC). The distance between the surface of the articular cartilage and the tidemark was defined as the thickness of the hyaline cartilage (HC), whereas the thickness of the CC was defined as the distance between the tidemark and the subchondral bone plate (SBP). The thickness parameters mentioned above was measured under 10 × magnification. Tartrate-resistant acid phosphatase (TRAP) staining was also performed following a standard protocol (Sigma-Aldrich). A standard protocol was used to perform the immunohistochemical analysis. The sections were incubated with primary antibodies against matrix metalloproteinase 13 (MMP-13) (Abcam, ab39012, 1:100), collagen X (Col X, Abcam, ab58632, 1:100), phosphorylated Smad2/3 (pSmad2/3, Santa Cruz Biotechnology Inc., 1:40), osterix (Abcam, 22552, 1:500), and CD31 (Abcam, ab28364, 1:100) overnight at 4°C. Subsequently, a horseradish peroxidase-streptavidin detection system (ZSGB BIO) was used to detect the immunoactivity, followed by counterstaining with hematoxylin (ZSGB BIO). A histomorphometric measurement was performed on the entire tibial subchondral bone using an Olympus DP26 microscope, and the quantitative analysis was conducted in a blinded way using cellSens software (Olympus, Int.). The total and positively stained chondrocyte numbers were calculated in the entire articular cartilage for MMP-13 and Col X analysis, whereas the pSmad2/3- and osterix-positive cells in the subchondral bone were counted in three views per specimen for the analysis. The histologic score of OA in medial tibial plateau was calculated as described by Glasson et al. (2010).

### Micro-computed tomography (μCT) analysis

The entire right knee joint of the mice was dissected with the soft tissue removed, and then fixed in 10% buffered formalin overnight. Then, the specimen were scanned by micro-computed (μCT, Skyscan 1176). The images were reconstructed (NRecon, v1.6), analyzed (CTAn, v1.9), and the data were analyzed further using three-dimensional (3D) model visualization (CTVol, v2.0). The micro-CT scanner was set at a voltage of 50 kVp, filter of 0.5 mm AI, and resolution of 9 μm/pixel. The sagittal view of the entire medial compartment of the tibial subchondral bone was used for the 3D histomorphometric analysis and the structural parameters analyzed were the trabecular thickness (Tb.Th), bone volume/total tissue volume (BV/TV), and Tb. pattern factor (Tb.Pf).

### μCT based microangiography

After the mice were euthanized, the thoracic cavity of the mice was opened and a needle was inserted into the left ventricle, through which the vascular circulation system was flushed with 0.9% normal saline containing heparin sodium of 100 μ/ml. Then the vascular circulation system was flushed with 10% neutral buffered formalin, followed by the radiopaque silicone rubber compound containing lead chromate (Microphil MV-122, Flow Tech). The mice were stored at 4°C overnight and then the knee joints were harvested and fixed in 10% neutral buffered formalin for 4 d. Then the specimen were decalcified in a rapid acid decalcifier solution (Rapid Cal Immuno, ZSGB-BIO) for 3 d in order to facilitate image thresholding of the vasculature from the surrounding tissues. μCT imaging system (μCT, Skyscan 1176) were used to obtain images and the μCT scanner was set at a voltage of 45 kVp, filter of 0.2 mm AI, and resolution of 9 μm/pixel.

### Statistics

The data are presented as the mean ± standard deviation (*SD*). One-way analysis of variance (ANOVA) with a significance level of 0.05 was used to determine whether the difference among different groups was statistically significant. The statistical package for the social sciences (SPSS) 22.0 (SPSS Inc.) was used for all the data analysis.

## Results

### HF preserved articular cartilage following oral gavage administration

**Figure 1:**
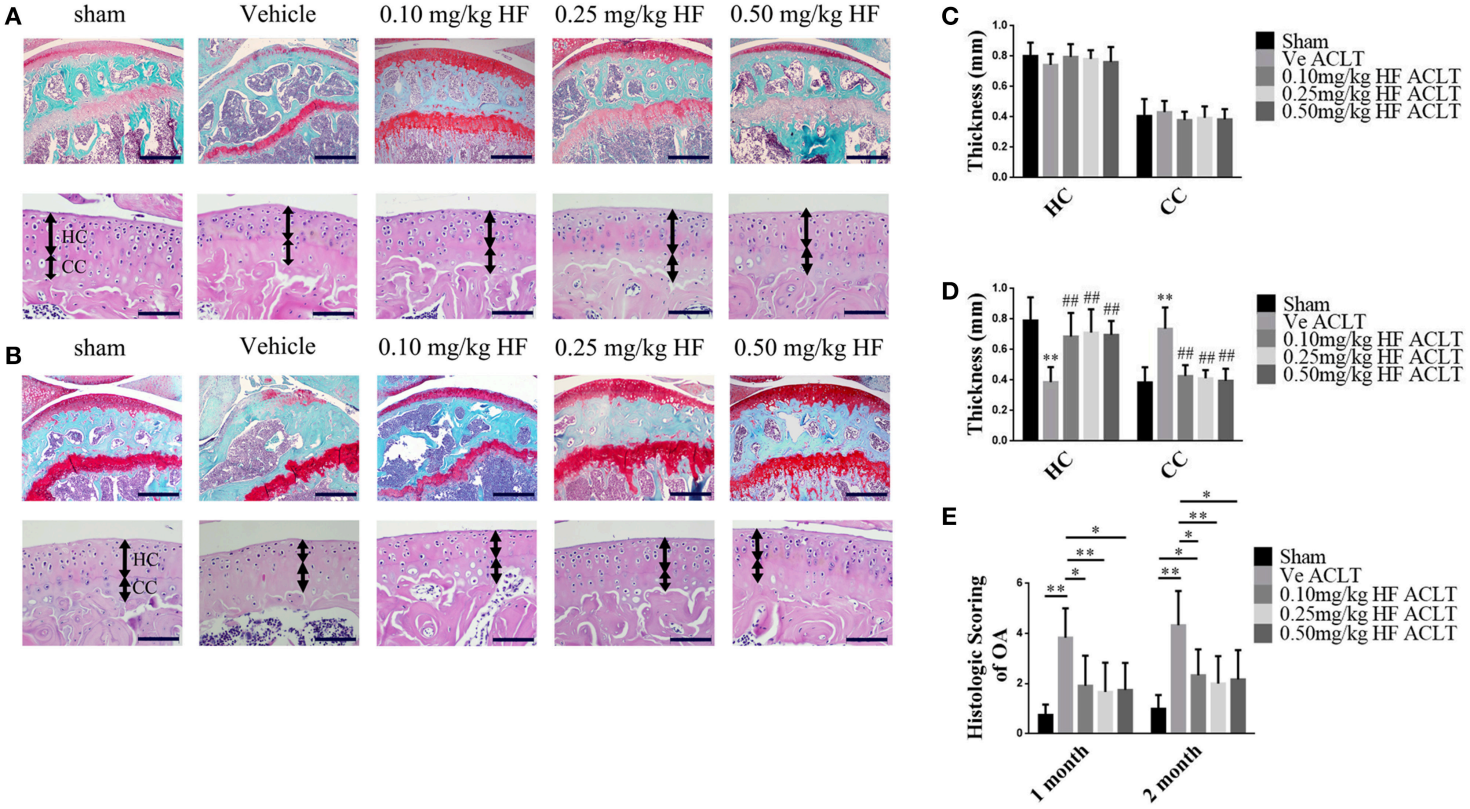
HF, by oral gavage, prevented articular cartilage degeneration after ACLT at 30 d **(A)** and 60 d **(B)**. Safranin O & Fast Green staining of sagittal sections of tibial medial compartment, where proteoglycan is in red and bone is in blue (top). Scale bar, 500 μm. H&E staining (bottom), where thickness of HC and CC are marked by double-headed arrows. Scale bar, 100 μm. HC and CC changes in thickness among different groups at 1 m **(C)** and 2 m **(D)** after sham surgery or ACLT. ^**^p < 0.01 compared to sham group, ##*p* < 0.01 compared to vehicle-treated ACLT group. **(E)** Histologic OA Score of articular cartilage at different time-points. ^*^p < 0.05 as denoted by bar, ^**^p < 0.01 as denoted by bar.

**Figure 2:**
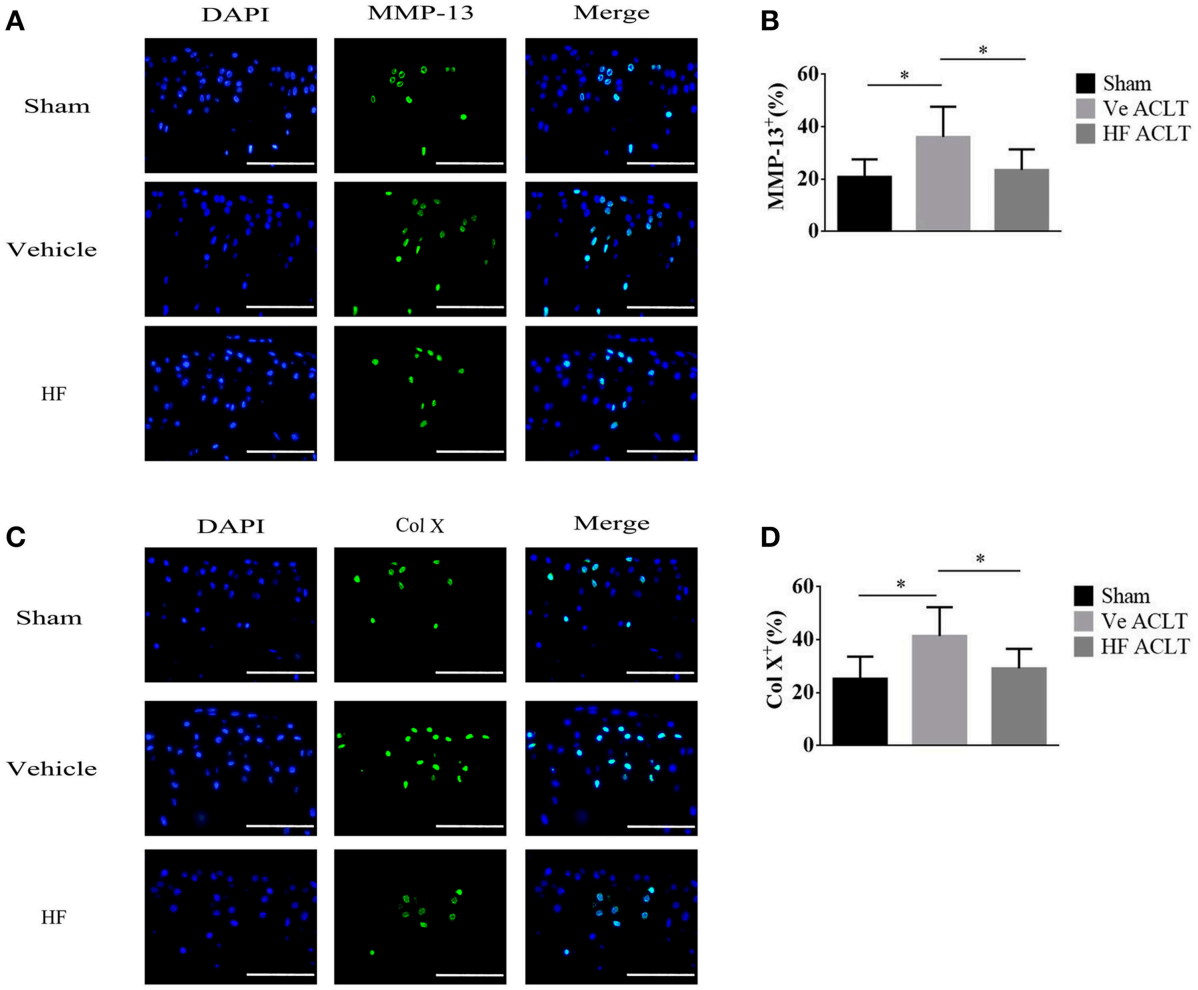
HF reduced the number of MMP-13 positive and Col X positive cells in articular cartilage at 30 d after ACLT. Immunofluorescent staining and quantitatively analysis of MMP-13 **(A,B)** and Col X **(C,D)**. Scale bar, 100 μm. ^*^p < 0.05 as denoted by bar.

The LD_50_ of HF was calculated as 3.7514 mg/kg, and the test doses used were 0.1, 0.25, and 0.5 mg/kg to investigate whether HF attenuated OA progression following oral gavage. An ACLT-induced OA mouse model was established and HF, which was administered at different doses every other day, protected the articular cartilage. Specifically, the Safranin O and Fast Green staining indicated that the proteoglycan loss in the HF-treated group was comparable to that of the Sham group (Figures 1A,B). Histologic score of OA in medial tibial plateau was performed to quantitatively assess the severity of cartilage degeneration. The vehicle-treated ACLT group showed almost twice the score of HF-treated ACLT groups at 1 and 2 months post-operatively (Figure 1E). Similarly, H&E staining showed that the thickness of the CC in vehicle-treated ACLT group was 1.91 times the thickness in sham group at 2 months post-operatively. However, no statistically significance was found in the thickness of HC and CC among sham group and HF-treated ACLT groups (*p* > 0.05) (Figures 1C,D). These results indicate that the three different dosages of HF also attenuated OA progression. HF of 0.25 mg/kg showed maximal effects, although the difference among the three dosage groups was not statistically significant. To validate whether HF itself would cause detrimental effect to the joint, the histologic score was compared and the thickness of HC and CC between sham group and sham + 0.25 mg/kg HF group was assessed. The sham + 0.50 mg/kg HF group was added to further investigate whether higher dosage of HF would harm the joint. As a result, the difference in the histologic score and thickness of HC and CC was not statistically significant among the three groups (*p* > 0.05) (Supplementary Figure 1). Therefore, HF of 0.25 mg/kg was used in the further analysis. The immunostaining results revealed that the number of MMP-13 positive cells was higher after ACLT (36.10 ± 11.53%) when compared with Sham group (20.88 ± 6.69%). After transecting the ACL, the number of MMP-13 positive cells was comparable to that of Sham group with use of HF (23.54 ± 7.82%) (Figure 2). The number of Col X positive cells increased following transecting the ACL (41.41 ± 10.89%). However, HF abrogated this phenomenon (29.25 ± 7.33%) to the level comparable to Sham group (25.26 ± 8.37%) (*p* > 0.05) (Figure 2). These results suggest that HF played a protective role against articular cartilage degeneration.

### HF maintained subchondral bone microarchitecture

**Figure 3:**
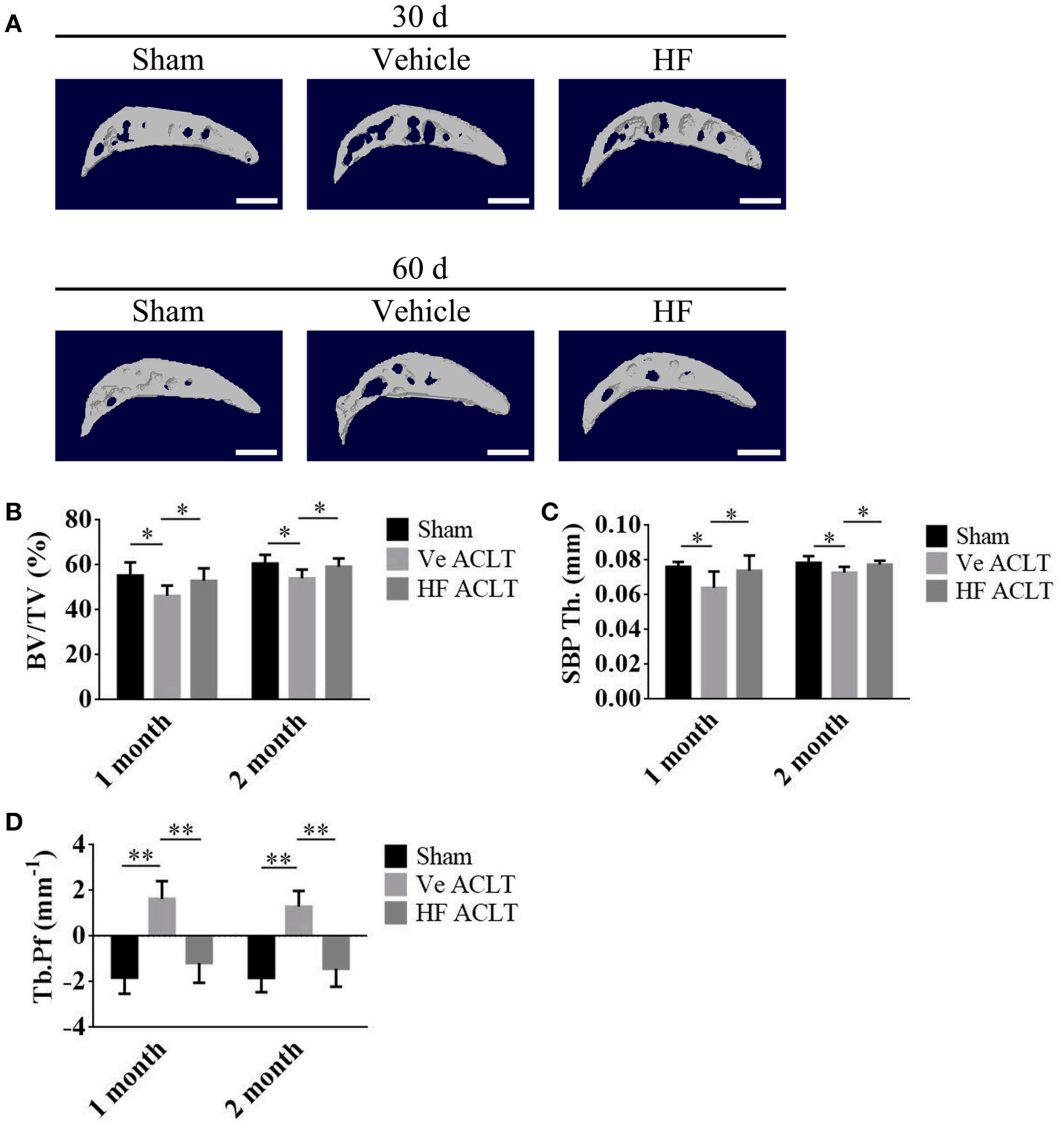
HF maintained normal subchondral bone microarchitecture after ACLT. **(A)** 3D micro-CT image of tibial subchondral bone medial compartment in sagittal view at 30 and 60 d after sham surgery or ACLT. Scale bar, 500 μm. **(B–D)** Quantitative analysis of micro-CT parameters of tibial subchondral bone: bone volume fraction (BV/TV) **(B)**, thickness of subchondral bone plate (SBP.Th.) **(C)** and trabecular pattern factor (Tb.Pf) **(D)**. ^*^p < 0.05 as denoted by bar, ^**^p < 0.01 as denoted by bar.

This study further investigated whether the protective effect of HF on the articular cartilage is associated with its potential effects on the subchondral bone. Micro-CT was used to analyze the subchondral bone structure. HF improved the subchondral bone microarchitecture (Figure 3A). The ratio of BV to TV was 55.34 ± 5.78 in the Sham group and it decreased substantially to 46.22 ± 4.58 in the vehicle-treated ACLT group. The effect of decreased BV/TV after ACLT was abrogated in the HF-treated group with the BV/TV of 52.99 ± 5.59 at 1 month post-operatively. Similar findings were also noticed at 2 months post-operatively, where BV/TV were decreased from 60.61 ± 3.93 in Sham group to 54.03 ± 3.87 in the vehicle-treated ACLT group and HF maintained it to 59.21 ± 3.75 (Figure 3B). The SBP and articular cartilage interact. Compared with the Sham group, the SBP thickness (SBP.Th.) was decreased from 0.0762 ± 0.0026 and 0.0784 ± 0.0039 mm to 0.0640 ± 0.0093 and 0.0728 ± 0.0034 mm in the vehicle-treated ACLT group at 1 and 2 months post-operatively. HF maintained the thickness of SBP to 0.0739 ± 0.0088 and 0.0774 ± 0.0023 mm with ACLT at 1 and 2 months post-operatively (Figure 3C). The Tb.Pf, with higher value indicating disruption of the connectivity and microarchitecture of the subchondral trabecular bone, was increased from −1.81 ± 0.72 and −1.83 ± 0.62 mm^−1^ in sham group to 1.63 ± 0.78 and 1.30 ± 0.69 mm^−1^ in the vehicle-treated ACLT group at 1 and 2 months post-operatively. Application of HF orally maintained the value of Tb.Pf to −1.17 ± 0.87 and −1.42 ± 0.79 mm^−1^ at 1 and 2 months respectively (Figure 3D). These results showed that in the vehicle-treated ACLT group, BV/TV and SBP.Th. was decreased while Tb.Pf was increased when compared with sham group and these effects were abrogated by using HF. Together, these data suggest that de novo aberrant bone formation of subchondral bone after ACLT could be prevented by the administration of HF via oral gavage.

### HF coupled subchondral bone remodeling and inhibited aberrant angiogenesis

**Figure 4:**
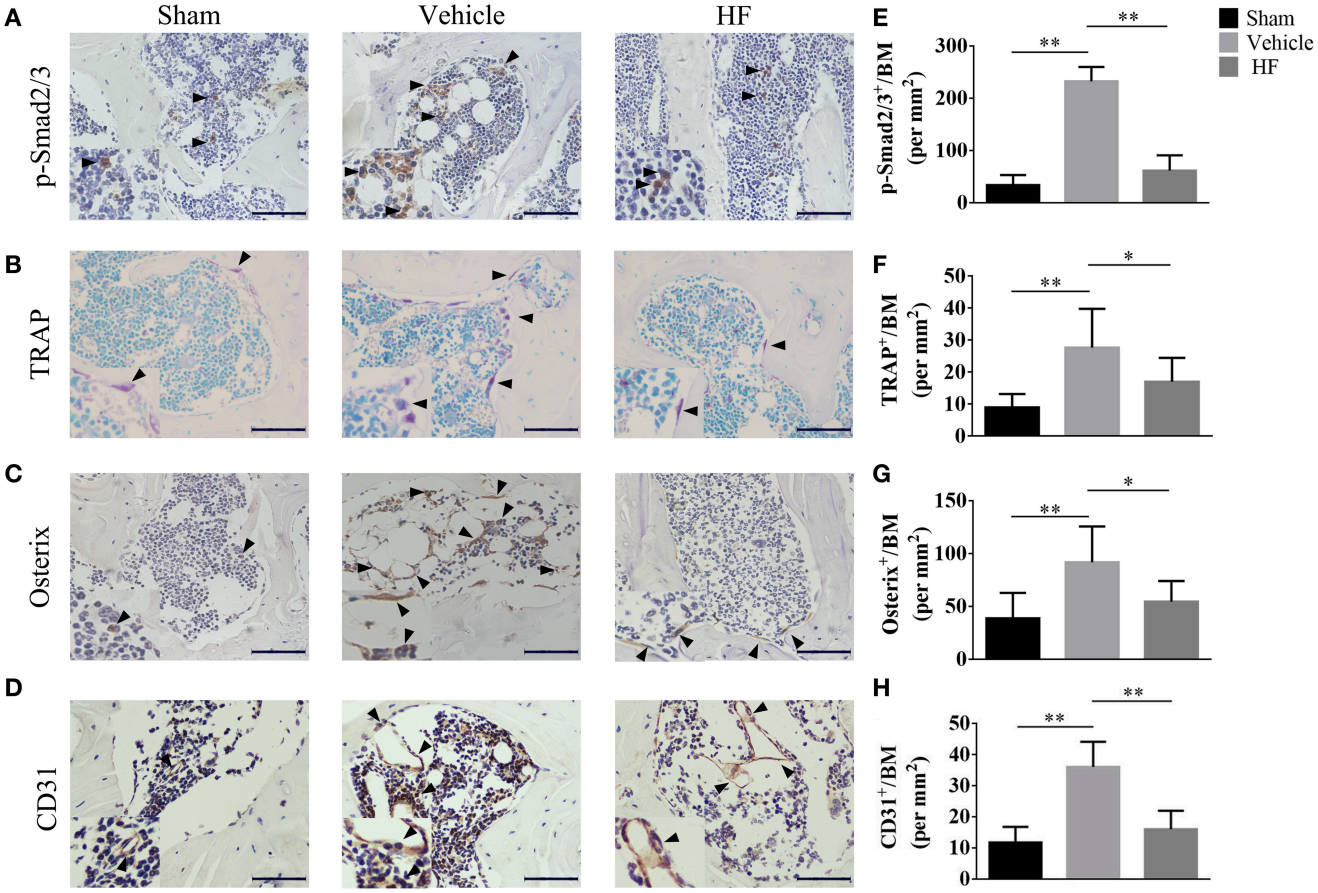
HF coupled subchondral bone remodeling and inhibited aberrant angiogenesis. Immunostaining and quantitative analysis of number of p-Smad2/3 positive cell **(A,E)**, TRAP-positive osteoclasts **(B,F)**, Osterix-positive osteoblast progenitors **(C,G)** and CD31 positive cells **(D,H)** in tibial subchondral bone at 30 d after sham surgery or ACLT. 100 μm. ^*^p < 0.05 as denoted by bar, ^**^p < 0.01 as denoted by bar.

**Figure 5:**
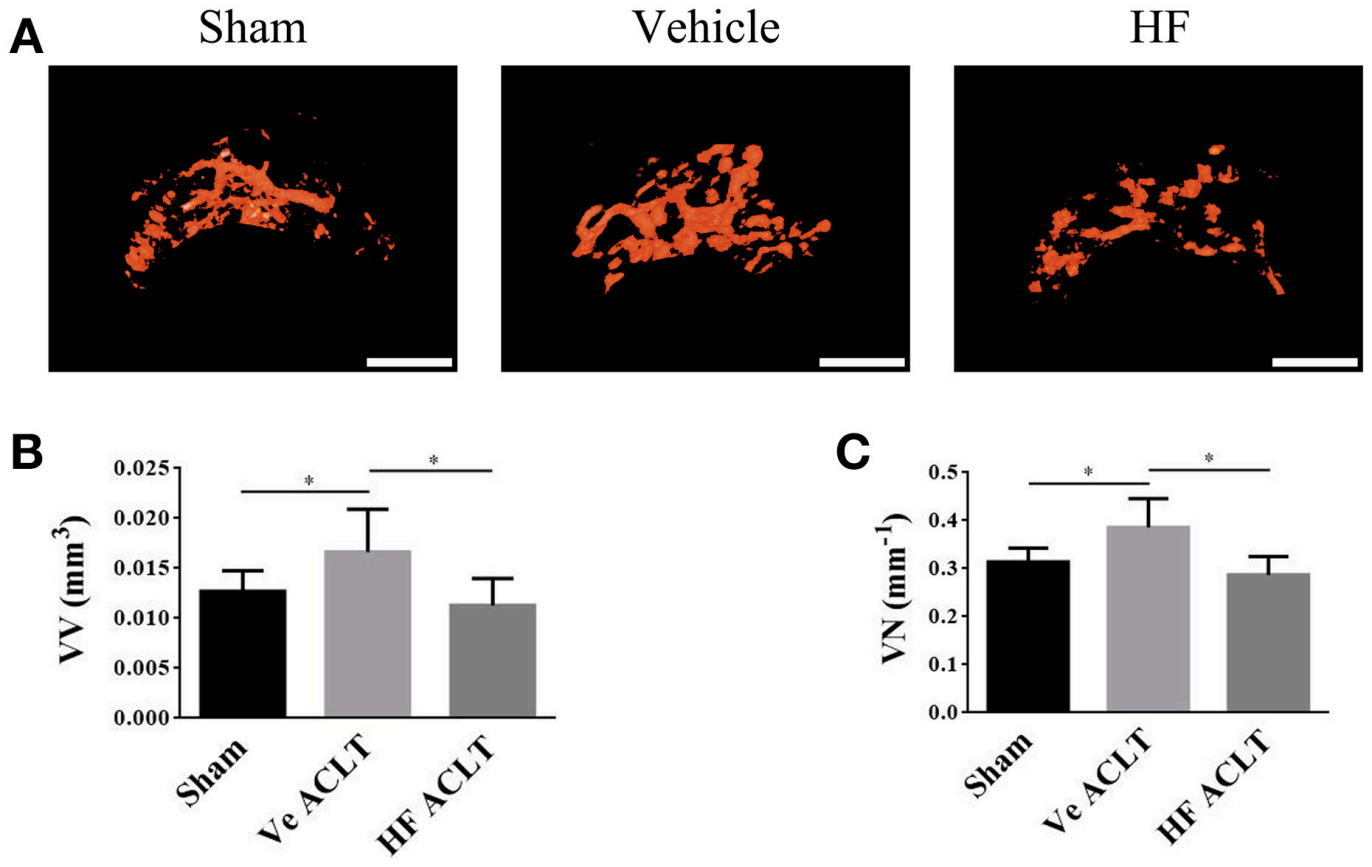
HF maintained the angiogenesis in subchondral bone. 3D μCT based microangiography of subchondral bone in medial tibial compartment **(A)** and quantitative analysis of vessel volume **(B)** and vessel number **(C)** in subchondral bone at 30 d after sham surgery or ACLT. Scale bar, 500 μm. ^*^p < 0.05 as denoted by bar.

The immunohistochemistry analysis was performed to investigate the potential mechanism underlying the effect of HF on the subchondral bone microarchitecture. It was found that the number of p-Smad2/3 positive cells was increased to 232.32 ± 27.61/mm^2^ significantly in the vehicle-treated ACLT group, whereas the difference in the number of p-Smad2/3-positive cells between Sham group (33.87 ± 19.29/mm^2^) and HF-treated ACLT group (61.62 ± 29.15/mm^2^) was not statistically significant (*p* > 0.05) (Figures 4A,E). Moreover, the number of TRAP-positive osteoclasts, which indicate the severity of bone resorption, was higher in the vehicle-treated ACLT group (27.67 ± 12.14/mm^2^) than Sham group (9.00 ± 4.19/mm^2^) and this effect was abrogated by HF to a level of 17.00 ± 7.48/mm^2^ (Figures 4B,F). Furthermore, HF not only affected the number of osterix-positive osteoprogenitors, but also maintained its location. Specifically, the number of osterix-positive osteoprogenitors was increased in the vehicle-treated ACLT group (91.75 ± 33.94/mm^2^) when compared with sham group(38.79 ± 24.09/mm^2^), and this effect was attenuated by HF to a level of 54.55 ± 19.51/mm^2^. Furthermore, HF also maintained the location of most osterix-positive cells to the bone surface, instead of the bone marrow, as was observed in the vehicle-treated ACLT group (Figures 4C,G). These results suggest that HF restored bone remodeling by inducing coupling bone resorption and formation. The effect of HF on vessel formation in subchondral bone was also investigated in this study. The results showed that the number of CD31 positive endothelial cells were more than three times higher in the vehicle-treated ACLT group (36.15 ± 8.09/mm^2^) when compared with sham group (11.90 ± 4.99/mm^2^) and HF downregulated its value (16.16 ± 5.92/mm^2^) to a level comparable to sham group (Figures 4D,H). Moreover, results from μCT based microangiography showed that the volume of vessels in subchondral bone was bigger than Sham group (0.0127 ± 0.0020 mm^3^) after ACLT (0.0166 ± 0.0042 mm^3^). This phenomenon was inhibited in HF ALCT group with the value of VV as (0.0113 ± 0.0027 mm^3^) (Figure 5B). Not only the volume of vessels in subchondral bone, but also the number of vessels in subchondral bone was increased in the vehicle-treated ACLT group (0.3860 ± 0.0601/mm) when compared with sham group. However, the difference in the VN between Sham group (0.3141 ± 0.0288/mm) and HF ACLT group (0.2874 ± 0.0377/mm) was not statistically significant (*p* > 0.05) (Figure 5C). Altogether, these results showed that HF significantly reduced the number of p-Smad2/3 positive cells, TRAP-positive osteoclasts and osterix-positive osteoprogenitors when compared with vehicle-treated group. HF also relocated the majority of osterix-positive osteoprogenitors to the bone surface, instead of subchondral bone marrow in the vehicle-treated group. Besides, HF inhibited the aberrant angiogenesis and restored the volume and number of vessels in subchondral bone to a level comparable to that of sham group (Figure 5).

## Discussion

Articular cartilage and subchondral bone function as a unit in the joint and homeostasis of the articular cartilage relies on its interplay with the subchondral bone (Burr and Gallant, 2012). In this study, an unstable, mechanical-loading OA model was established by transecting the ACL. Furthermore, the established model was used to show that HF administered by oral gavage re-established coupled bone remodeling, reduced aberrant angiogenesis, maintained the subchondral bone microarchitecture, preserved the articular cartilage from degeneration and thus slowed OA progression.

The microarchitecture of the subchondral bone changes with the onset of OA, which may precede the articular cartilage degeneration (Zhen et al., 2013). Micro-CT is used to detect subtle changes in subchondral bone. In the present study, it was found that the BV/TV and SBP.Th. were decreased, whereas the Tb.Pf was increased in the vehicle-treated group compared with values in the Sham group. These findings are in agreement with those of previous studies (Botter et al., 2006; Sniekers et al., 2008; Xie et al., 2016). However, the value of these parameters was comparable between the Sham group and HF-treated ACLT groups, indicating that HF maintained the properties of the subchondral bone.

Bone remodeling of the subchondral bone is the major step in mechanical loading changes, which involves bone matrix degradation and formation by osteoclast and osteoblast (Castañeda et al., 2012). Bone resorption and formation occur at specific region following well-defined cascades of events. Osteoclasts resorb the bone at the site of the bone surface during normal bone remodeling, followed by bone formation. It has been reported that TGF-β is an important coupling factor of bone resorption and formation, which were buried in the bone mineral matrix. TGF-β was released and activated from bone mineral matrix following osteoclast bone resorption. The active TGF-β will direct the mesenchymal stem cells (MSCs) to form the new bone exactly where bone resorption occurs. Osterix-positive osteoprogenitor from MSCs are the precursor cells of osteoblasts. Osteoblasts and their progenitor cells are mainly located at the bone resorption site on bone surface during normal physiological bone remodeling process. However, abnormal mechanical loading results in excessive release and activation of TGF-β from bone mineral matrix in subchondral bone owing to increased bone resorption (Zhen et al., 2013; Shen et al., 2014). Dysregulation of TGF-β alters MSCs recruitment and their fate, uncoupling bone remodeling. Specifically, the excessively released TGF-β mediate further differentiation of MSCs into osteoblast precursors and lead to the commitment of osteoprogenitors in-situ in bone marrow cavities instead of bone surface. The clustered bone marrow osteoprogenitors will lead to osteoid islets in the subchondral bone marrow, visualized as bone marrow lesions from MRI and identified as a prognostic factor of OA progression (Suri and Walsh, 2012), further resulting in alteration of SBP.Th. and advancement of CC into the overlying HC (Bullough, 2004; Suri and Walsh, 2012). In this study, it was found that HF administered by oral gavage, prevented abnormal bone resorption and aberrant bone formation. Besides, the activity TGF-β, which were indicated by the number of p-Smad 2/3 positive cells in subchondral bone was decreased to a level comparable to that in Sham group with HF after ACLT. The possible underlying mechanism of rescuing uncoupled bone remodeling with HF involves a decrease in the release and activation of TGF-β from the bone mineral matrix because HF reduced abnormal bone resorption, thus further inhibited osteoid islets formation.

Adequate blood supply is critical to bone tissues and bone formation is usually coupled with vessel formation (Portal-Núñez et al., 2012). Blood circulation can supply nutrients, oxygen, minerals that required during osteogenesis and it can carry away the metabolic waste (Percival and Richtsmeier, 2013). Aberrant vessel formation in subchondral bone is a known characteristic of OA (Lotz, 2012). Increasing the TGFβ signaling in endothelial progenitor cells can enhance vessel formation (Cunha and Pietras, 2011) Besides, TGFβ has also been demonstrated to promote angiogenesis via stimulating the paracrine machinery in MSCs (Guiducci et al., 2011). HF has been demonstrated to result in inhibitory effects on representative sequential events in the angiogenic cascade (Pines and Spector, 2015). It inhibits the diameter and length of vessels in tumor tissue (Gavish et al., 2002). CD31 is a classic marker of endothelial cells and it is used for evaluation of angiogenesis (Newman et al., 1990). In the present study, increased CD31 positive staining in subchondral bone was observed in the vehicle-treated ACLT group. Moreover, micro-CT based microangiography analysis showed that both the vessel volume and vessel number was increased after ACLT. These results confirmed the role of aberrant vessel formation in subchondral bone during the progression of OA. By way of oral gavage, HF abrogated the aberrant angiogenesis, maintained both the volume and number of vessels in subchondral bone. Previously it has been demonstrated that abrogation of increased TGFβ activity in subchondral bone can normalize the aberrant angiogenesis in subchondral bone during OA (Zhen et al., 2013). This study showed that HF reduced the number of p-Smad 2/3 positive cells and inhibited aberrant angiogenesis in subchondral bone, contributing to the maintenance of the subchondral bone microarchitecture. It is hypothesized that the profound effect of HF on normalization of angiogenesis in subchondral bone was likely primarily due to the inhibition of elevated TGFβ activity in subchondral bone and further *in-vitro* experiment are needed to validate the exact mechanism in the future.

The normalization of bone remodeling and angiogenesis in the subchondral bone preserved the overlying articular cartilage. Cartilage is composed of specialized chondrocyte cells that produce a large amount of collagenous extracellular matrix proteins including proteoglycan and type II collagen (collagen II). Proteoglycan and collagen II maintain the homeostasis of the articular cartilage, which keeps it intact. During the progression of OA, the chondrocytes hypertrophy and synthesized proinflammatory cytokines that contribute to their destruction (Luyten et al., 2006; Goldring and Goldring, 2010). Col X is a classic marker of hypertrophic differentiation of chondrocytes (von der Mark et al., 1992). MMP-13 is the primary collagenase synthesized by chondrocytes, which damages aggrecan and collagen II during OA. The histologic scoring system for OA in mice is used to quantitatively assess the severity of cartilage damage (Glasson et al., 2010). In the present study, it was discovered that advancement of the CC zone was blocked in the HF-treated ACLT group compared with that in the vehicle-treated ACLT group. It was also noticed that treatment with HF reduced the number of Col X and MMP-13 positive cells in the cartilage and lowered the histologic score more than that of the vehicle-treated ACLT group. These results demonstrate that HF rescued the homeostasis and integrity of the articular cartilage.

HF was investigated in clinical trials for the treatment of chronic graft-vs.-host disease and solid tumors and showed safe therapeutic efficacy following oral administration (Nagler and Pines, 1999; De Jonge et al., 2006). The results of this study may contribute to expanding its clinical application. The kinetics of drug uptake and distribution may differ dramatically between the various routes of administration and bioavailability following oral administration may be relatively lower. However, oral administration is a considerably more convenient route that is often more acceptable to patients, especially for those who need to repeatedly administer medications. In the present study, for the first time, it was shown that HF administered by oral gavage recoupled bone remodeling and inhibited aberrant angiogenesis in the subchondral bone in early-stage OA. More importantly, the articular cartilage, which is the primary concern in OA treatment, was preserved. Altogether, these results indicate that HF administered by oral gavage is an effective strategy for preventing OA progression.

## Author contributions

WM: Designed, performed the research and wrote the paper; BX: Performed the research and contributed reagents; HM: Designed the research and edited the paper; JL: Performed the research; BJ and ZZ: Analyzed the data; AA: Coordinated the research process; LC: Revised the paper and guided the research.

### Conflict of interest statement

The authors declare that the research was conducted in the absence of any commercial or financial relationships that could be construed as a potential conflict of interest.

## Supplementary material

Supplementary Figure 1 HF, by oral gavage, shows no obvious detrimental effect on the joint after sham operation at 30 d **(A)** and 60 d **(B)**. Safranin O & Fast Green staining of sagittal sections of tibial medial compartment, where proteoglycan is in red and bone is in blue (top). Scale bar, 500 μm. H&E staining (**A**, bottom, **B**, bottom), where thickness of HC and CC are marked by double-headed arrows at 1 m **(A)** and 2 m **(B)** after sham surgery. Scale bar, 100 μm. Changes of HC and CC in thickness among different groups at 1 m **(C)** and 2 m **(D)** after sham surgery. **(E)** Histologic OA Score of articular cartilage at different time-points.
