# Supplementary figures and images for: Halofuginone Attenuates Osteoarthritis by Rescuing Bone Remodeling in Subchondral Bone Through Oral Gavage
Source: Front Pharmacol. 2018 Mar 27;9:269. doi: 10.3389/fphar.2018.00269 (PMC5881118; doi:10.3389/fphar.2018.00269)

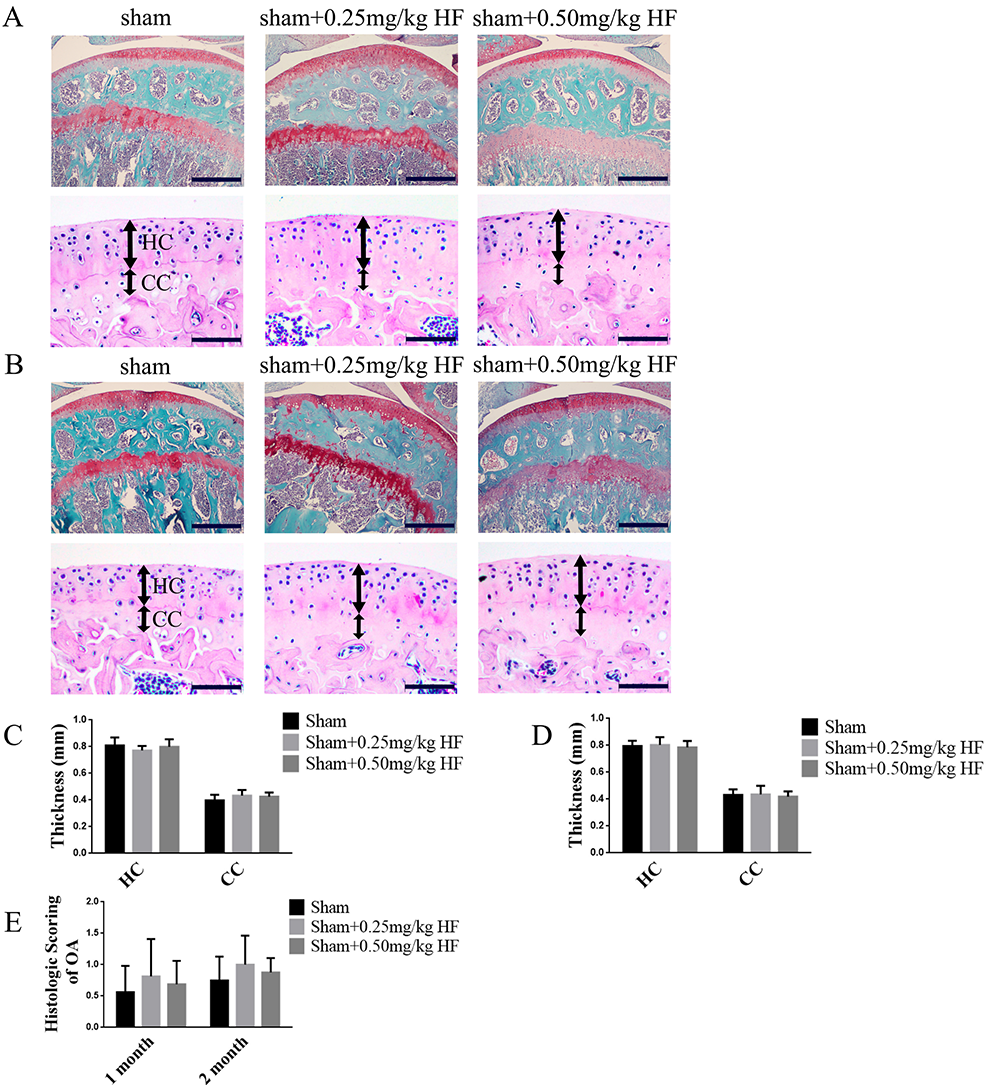

Supplement: Supplementary Figure 1 — HF, by oral gavage, shows no obvious detrimental effect on the joint after sham operation at 30 d (A) and 60 d (B). Safranin O & Fast Green staining of sagittal sections of tibial medial compartment, where proteoglycan is in red and bone is in blue (top). Scale bar, 500 μm. H&E staining (A, bottom, B, bottom), where thickness of HC and CC are marked by double-headed arrows at 1 m (A) and 2 m (B) after sham surgery. Scale bar, 100 μm. Changes of HC and CC in thickness among different groups at 1 m (C) and 2 m (D) after sham surgery. (E) Histologic OA Score of articular cartilage at different time-points. [file Image1.TIF]
